# In-Vitro Analysis of FeMn-Si Smart Biodegradable Alloy

**DOI:** 10.3390/ma15020568

**Published:** 2022-01-12

**Authors:** Ana Maria Roman, Victor Geantă, Ramona Cimpoeșu, Corneliu Munteanu, Nicoleta Monica Lohan, Georgeta Zegan, Eduard Radu Cernei, Iulian Ioniță, Nicanor Cimpoeșu, Nicoleta Ioanid

**Affiliations:** 1 Faculty of Materials Science and Engineering, Gh. Asachi Technical University from Iasi, 700050 Iasi, Romania; ana-maria.roman@academic.tuiasi.ro (A.M.R.); monica.lohan@yahoo.com (N.M.L.); iulian.ionita@academic.tuiasi.ro (I.I.); nicanor.cimpoesu@tuiasi.ro (N.C.); 2 Faculty of Materials Science and Engineering, University Politehn Bucuresti, Splaiul Independentei 313, 060042 Bucharest, Romania; victorgeanta@yahoo.com; 3 Faculty of Mechanical, “Gh. Asachi” Technical University from Iasi, 700050 Iasi, Romania; cornelmun@gmail.com; 4 Faculty of Dental Medicine, “Grigore T. Popa” University of Medicine and Pharmacy, 700050 Iasi, Romania; eduard-radu.cernei@umfiasi.ro (E.R.C.); nicole_ioanid@yahoo.com (N.I.)

**Keywords:** iron based biodegradable alloy

## Abstract

Special materials are required in many applications to fulfill specific medical or industrial necessities. Biodegradable metallic materials present many attractive properties, especially mechanical ones correlated with good biocompatibility with vivant bodies. A biodegradable iron-based material was realized through electric arc-melting and induction furnace homogenization. The new chemical composition obtained presented a special property named SME (shape memory effect) based on the martensite transformation. Preliminary results about this special biodegradable material with a new chemical composition were realized for the chemical composition and structural and thermal characterization. Corrosion resistance was evaluated in Ringer’s solution through immersion tests for 1, 3, and 7 days, the solution pH was measured in time for 3 days with values for each minute, and electro-corrosion was measured using a potentiostat and a three electrode cell. The mass loss of the samples during immersion and electro-corrosion was evaluated and the surface condition was studied by scanning electron microscopy (SEM) and energy dispersive spectroscopy (EDS). SME was highlighted with differential scanning calorimetry (DSC). The results confirm the possibility of a memory effect of the materials in the wrought case and a generalized corrosion (Tafel and cyclic potentiometry and EIS) with the formation of iron oxides and a corrosion rate favorable for applications that require a longer implantation period.

## 1. Introduction

A special class of degradable biomaterials is intended for temporary implants whose presence is necessary to heal diseased tissue [1]. These types of implants work based on the same principle, but applications for different physiological environments differentiate them. For example, in temporary cardiovascular applications, coronary stents must open a narrowed artery and keep it open until the blood vessel is healed by replacing old tissues with newly formed ones [2,3]. In the case of orthopedic applications, implants of this type heal a fractured bone, keep it sustained until a healthy bone tissue is formed to replace the implant, which should then degrade. For use in cardiovascular applications such as stents, these biodegradable metals have shown adequate properties and special purity of the metal following the process of obtaining them from metallurgy and electrodeposition. [4]. The classical methods of thermal and thermomechanical treatments also play an essential role in obtaining properties with a specific destination for medical applications.

To fulfill their function in the healing process and to be successful in application, the biodegradable materials used for coronary stents must have a balance between the mechanical properties and the degradation process [5]. The speed with which the degradation occurs is crucial to allow the stent to maintain its mechanical resistance long enough to be able to heal the diseased arterial vein. The healing period in this case can be between 6 and 12 months [6,7]. The speed with which the material degrades must be optimal to allow the waste resulting from the degradation to be eliminated from the body. Waste accumulation around the implant can be harmful and can cause other unwanted injuries. Some studies indicate a favorable period for complete degradation of a stent to be between 12–24 months after implantation [8].

Studies on biodegradable metals present different methods in material development and improvement of mechanical properties. In vitro and in vivo studies are performed to obtain an optimal rate of degradation. Data were recorded on Fe-Mn alloy coronary stents [9], WZ21 Mg gastrointestinal implant [10], and Mg implant for laryngeal micro-surgery [11].

Fe plays an important role in the breakdown of lipids, proteins, and DNA damage by producing reactive species following the Fenton reaction [12,13,14].

Following in vitro research, pure Fe has had a positive effect on the prevention of restenosis [12,15]. Another alloying element that can be associated with Fe is Mn. Excess Mn in the body has not been shown to be toxic. The alloying of Fe with Mn led to the production of new austenitic and some antiferromagnetic alloys, compatible with the magnetic field of MRI [16]. Fe-Mn alloys are influenced in the degradation process by increasing the corrosion rate given by Mn from the oxide layer formed. Zhang et al. [17] showed that the additional corrosion of the substrate is due to the Mn oxide present on the metal surface. Dargusch et al. [16] confirmed this in his paper, noting the uniform distribution of Mn oxide on the corrosion layer of the Fe-Mn alloy. Another important factor that could influence the rate of degradation is deformation. In the study by Heiden et al. [18], it was concluded that the rate of degradation of cold-rolled Fe-20Mn alloy was slower than that of the same cast alloy due to the more protective oxide layer formed on the metal surface. Hermawan et al. [19,20,21,22] has numerous studies on biodegradable Fe-Mn alloys, thus giving encouraging prospects for future studies in the design of new alloys based on Fe-Mn alloys. Several classes of new materials have been proposed, such as Fe-Mn-Pd alloys [23], Fe-Mn-(Co, C, Al, etc.) [24] as having a good degradation behavior and mechanical properties suitable for these types of implants. Specialist studies have shown the mechanism of Fe-Mn alloy degradation during dynamic degradation tests in the solution modified by Hank’s solution. Following in vitro tests, Fe-Mn alloys showed a better degradation rate, 220–240 µm/year, than in the case of pure Fe [25].

The basic property of SMA (shape memory alloys) is that when thermally or mechanically activated they have the SME and the pseudo-elastic effect. If we add to these materials with special properties noted above, good resistance to corrosion and bending, and compatibility with magnetic and biological resonance, we obtain special materials that will undoubtedly be the best candidates in choosing materials for different medical applications. The addition of Si in the alloys of the Fe-Mn system leads to the appearance of the SME, as previously demonstrated [26,27]. B. Liu et al. in 2010 obtained favorable results following studies conducted on FeMn-Si alloys as candidates for biodegradable alloys. The important aspects that raise the issue of investigations on these alloys for applications in biodegradable implants are related to the microstructure, mechanical properties and SME, biocompatibility, and good degradation rate. The SME of FeMn-Si alloys formed by ε-martensite and γ-austenite phases was clearly shown. Also, with the addition of Si, an increase in the content of the γ-austenite phase was observed [28].

In this article, the behavior of the corrosive environment in the cast and wrought state of FeMnSi alloy was presented with emphasis on degradation properties of the materials. SEM, EDS, and EIS results are given to highlight the surface state of the samples after contact with Ringer’s solution. The enhancement of corrosion rate with addition of Si to an FeMn alloy is expected and differences of corrosion rate between cast and wrought samples were observed.

## 2. Materials and Methods

An experimental alloy, FeMn-Si was obtained from high purity materials in a vacuum Arc Melting Facility MRF ABJ 900 (University Politehnica Bucharest, Bucharest, Romania), which ensured the melting of metallic materials in Ar-controlled atmosphere after preemptive working chamber up to 10^−5^ mbar by using a non-consumable throttle tungsten mobile electrode. The re-melting process, repeated five times, occurred in an induction furnace (Inductro, Bucharest, Romania) in ceramic crucible at “Gheorghe Asachi” Technical University in Iasi. The ingot was wrought until it was a 1 mm sheet, using a hot rolling equipment with the sample heated to 1100 °C and 5 reduction passes. The samples analyzed in this article were in cast and wrought states (C and W), both heat-treated through solution water quenching (heated to 1100 °C, maintained for 5 min for temperature homogenization and cooled in water + ice). For experimental tests, the specimens were mechanically polished with Al_2_O_3_ suspension solution (2–5 µm) after metallographic grinding with paper disks with 160–4000 MPi granulation. The cleaning of the surface was done with ethyl-alcohol for 30 min and the microstructure was highlighted by chemical etching using Nital 2% solution [29].

Two sample fragments were cut, weighing less than 50 mg, for DSC experiments (Partner digital balance). A differential scanning calorimeter type DSC 200 F3 Maya (NETZSCH, Selb, Deutschland) was used, with sensitivity: <l W, temperature accuracy of 0.1 K, and enthalpy accuracy generally <1%. The calibration was done according to the standards with Hg, Bi, In, Sn, and Zn. Temperature scans were performed with the following temperature program: cooling from room temperature to −50 °C, heating from −50 °C to 200 °C, and cooling to room temperature. The cooling and heating rate was 10 K/min. All experiments were performed under an Ar protective atmosphere. NETZSCH’s Proteus software version 4.8.5 was used to evaluate the DSC thermograms resulting from cooling and heating using the tangent method for determining critical temperatures and a rectilinear baseline for dissipated/absorbed heat.

The samples were subject to immersion tests in Ringer’s solution (one of the first laboratory solutions of salts in water shown to greatly prolong the survival time of excised tissue; the solution contains calcium, potassium, and sodium chlorides, and sodium bicarbonate in the concentrations in which they occur in body fluids) at 37 °C, for 1, 3, and 7 days to analyze the interaction between the metallic materials and an electrolyte solution. The samples were weighed using a AS220 Partner analytical balance (Partner Co., Bucharest, Romania), before immersion, after immersion, and after an ultrasound cleaning stage (ultrasonic bath, 60 min in technical alcohol). The solution pH values were recorded with an Arduino set-up each minute and the variations were analyzed to establish the chemical reactions that occurred during the contact of the samples with the solution. Chemical composition of the surface was established with an EDS detector, Bruker X-flash, Mannheim, Germany Scanning electron microscopy (SEM, VegaTescan LMH II, SE detector, 30 kV, Brno—Kohoutovice, Czech Republic) was used to analyze the experimental materials structure and the state of the surface after immersion tests and electro-corrosion. X-ray diffraction tests were made with an Expert PRO-MPD system, (XRD, Panalytical, Almelo, The Netherlands type, Cu-X-ray tube (Kα-1.54°)).

The corrosion behavior of the samples was studied by comparing the method applied in the case of Fe-based alloys, using Ringer’s solution with standard composition (chemical composition for Ringer’s solution: 1000 mL contains: sodium chloride 8.6 g, calcium chloride × 6H_2_O 0.5 g, potassium chloride 0.3 g, distilled water up to 1000 mL) as the liquid medium. The VoltaLab-21 potentiometer (Radiometer, Copenhagen, Denmark) was used to determine the corrosion resistance by analyzing linear and cyclic curves in Ringer’s electrolyte solution, and the acquisition and processing of data was done with the Volta Master 4 package, version 6.0. A cell was used to expose the sample (working electrode) to Ringer’s solution, with an auxiliary Pt electrode and one saturated with calomel. The samples were isolated with Teflon, so only one area was exposed to the electrolyte, an area of 0.78 cm^2^. The solution was aerated permanently with a magnetic stirrer to remove gas bubbles from the metal surface following the removal of hydrogen. The potential-dynamic polarization test recorded data on electrode behavior. Through the polarization mechanisms of the direct current, approximate information was obtained about the corrosion speed of the working electrode (Fe-based samples), the type of surface corrosion (generalized or pitting), formation and stability of the passivation layer, anodic reactions (oxidation), or cathodic reactions (reduction).

The authors chose the following coordinates corresponding to the function: for the current density [mA/cm²] the potential [V], a variation that allows the accentuation of the corrosion potential (E_corr_) and the corrosion current (J_corr_). The temperature of the experiments was room temperature (±24 °C) and the potential was recorded (line graphs were recorded at a scan rate of 1 mV/s and cycle graphs at a scan rate of 10 mV/s). For the accuracy of the results the experiments were repeated four times. Corrosion current values helped to obtain the instantaneous corrosion rate: V_corr_ (µm/year) [30].

## 3. Results

Microstructural, chemical, and thermal characteristics of the new chemical composition SMA were characterized using scanning electron microscopy (SEM), energy dispersive spectroscopy (EDS), and differential scanning calorimetry (DSC). The corrosion behavior of the alloy was evaluated through pH variation of electrolyte solution, immersion tests, linear and cyclic potentiometry, and EIS experiments.

### 3.1. Experimental FeMnSi Materials Analysis

The experimental materials were mechanically ground to remove oxides from the surface and cleaned in an ultrasound bath in technical alcohol. The chemical composition of the samples (cast and wrought state) was determined in five different areas of the surface, and the average values are shown in Table 1. Standard deviations of the elements show a homogeneous chemical composition of the material that will confirm the same properties of the material for the entire volume. Good chemical and structural homogeneity are essential for biodegradable materials and for SMA properties [31].

**Figure 1:**
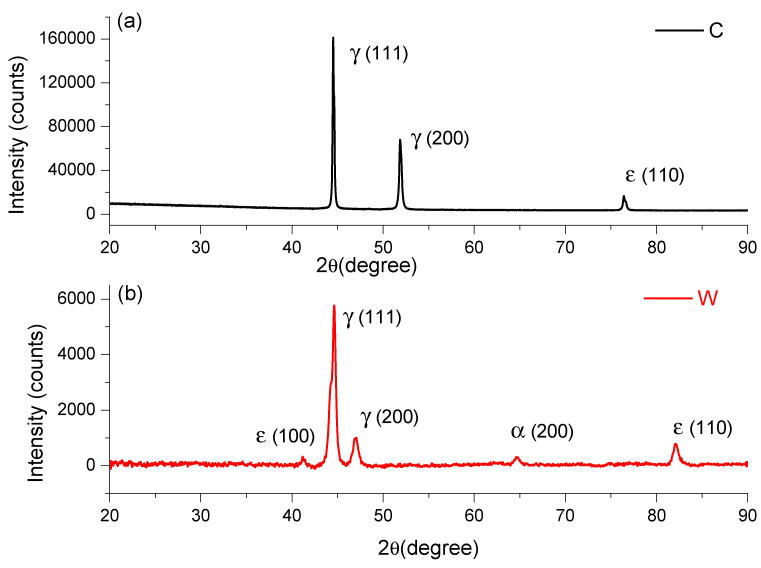
XRD spectra of FeMnSi: (**a**) cast and (**b**) wrought.

The chemical composition of the FeMnSi samples that was obtained leads to the appearance of the SME [32]. The main alloying element required by these FeMnSi alloys is manganese, with two roles in the thermodynamic stability of the phases. First, manganese stabilizes γ-austenite with the FCC structure being a phase that occurs at high temperatures in the case of iron. At normal pressure, manganese plays the role of stabilizing the ε phase with the HCP type structure, the thermodynamically stable phase only at high pressure for pure iron. The thermodynamic equilibrium temperature between phases γ- and ε- is close to room temperature, too low for the diffusion of atoms; phase ε is formed as martensite under cooling or loading. In Figure 1, XRD peaks of FeMnSi in cast, analyzed in [29] and wrought state, are given.

On the rolled sample we observed and identify five main phases (see Figure 1), at the following angles: 41.23619°, 44.65729°, 46,984.93°, 64.69378°, and 82.12412°. It is known that iron has several allotropic forms, specifically (at normal pressure): α-Fe with a cube structure with centered volume, stable up to 912 °C; γ-Fe with a cube-type structure with centered faces, stable up to at 1394 °C; Pand δ-Fe with a cube structure with centered volume, stable up to 1538 °C (melting temperature). Iron-based alloys will have phases in the structure with similar structures, but depending on the alloying elements, the field of these phases is modified. In addition to these phases, new phases such as intermetallic compounds or carbides may appear in the iron alloy systems, which will lead to the appearance of new metallographic constituents. Phase diagrams give indications of the phases that can occur in various alloy systems, especially binary or ternary. The higher the number of alloying elements and the more diverse the chemical composition, the more phases can occur in the alloy system. 

Given the chemical composition of the analyzed alloys (Mn is the second alloying element as a percentage), the analysis of the equilibrium diagram of the Fe-Mn binary system shows that at the mass concentration of this element, at room temperature, there are an α-Fe phase and a γ-Fe phase, solid manganese iron solutions that may have similar structures but with different network parameters. Manganese is an alloying element that increases the range of the γ-Fe phase. Instead, both silicon and aluminium are alloying elements that increase the range of the α-Fe phase. From the analysis of the phase diagram and of the considerations stated above, it can be considered as a working hypothesis that the two alloys will have in the structure either the α-Fe type phase or both α-Fe and γ-Fe type phases. In this case, after the rolling deformation process, the peak of ε (110) at 82° presented an increase in the wrought state compared to cast state and, likewise, the appearance of the peak ε (100) at 41.23619°.

The transformation γ → ε depends on SME, so pretensioning, annealing treatment, thermomechanical training, and deformation temperature influence the FeMnSi SME [33]. Sato et al. studied Fe-30Mn-1Si alloy single crystals that showed a large shape recovery strain. [34]. In the FeMnSi system, the most indicated concentration ranges are 14–33% mass for Mn and 4–6% mass for Si, respectively. The composition is chosen for the beginning of the martensitic transformation, Ms, temperatures close to room temperature. For SMA, different chemical compositions based on FeMnSi have been proposed by partially replacing Mn with the elements Cr, Ni, Cu, Al, etc. [35,36,37,38]. The ideal proportion of the alloy components is made according to the temperature Ms of the transformation γ → ε and the stability of austenite compared to ά-martensite. Studies show that a small number of interstitial elements, such as C and N, strongly stabilize the γ-austenite phase and reduce the concentration of Mn when they are dissolved in the γ phase. [39]. The properties of the material can be improved by alloying with new elements. The new properties obtained refer to aspects related to the increase in corrosion resistance, mechanical resistance, the formability, and the decrease in the production cost. To conduct the phase transformation and to obtain the SME, the appropriate concentrations of the alloy can be calculated with the equations for the Gibbs free energy difference between the phases γ- and ε- and the equations for the temperature Ms of the transformation γ → ε [40]. Alloying with Si strengthens the matrix to suppress the dislocation slip and helps the martensitic transformation γ → ε by decreasing the energy of the stacking defect [41,42]. An important property such as that related to magnetism affects the phase transformation in its transformation into ε-martensite. Silicon lowers the magnetic transition temperature of FeMnSi-based alloys to sub-zero temperatures and leads to the martensitic transformation γ → ε at room temperature. Another influence that Si has is that it can lead to the short-term ordering of atoms to improve the martensitic transformation ε → γ [43]. Another factor that raises the reversibility of the inverse transformation is the improved coherence between the γ and ε networks with the addition of silicon. An interesting aspect would be that despite such complicated factors, the optimal amount of Si needed to reach the best shape recovery strain is always between 4 and 6% by mass.

**Figure 2:**
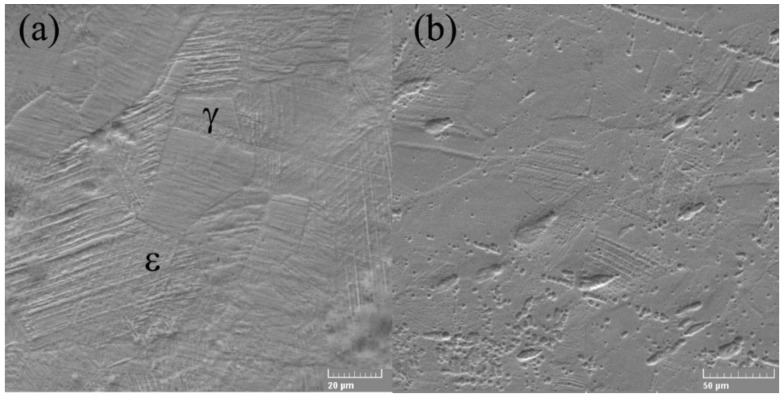
SEM micrographs of FeMnSi SMA (**a**) cast and (**b**) wrought.

The SEM electron microscope was used to investigate the microstructures of alloys in both states (cast and rolled) at high amplification. A detailed analysis of the microstructure did not show the presence of ά martensite, which is usually present in the lenticular form, respectively, Figure 2a,b.

**Figure 3:**
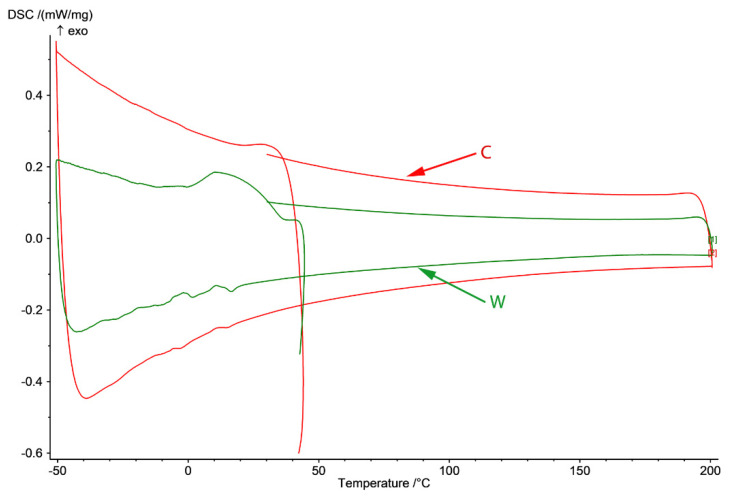
DSC diagram of C and W samples in −50 to 200 °C domain (heating from 25 to 200 °C and cooling to −50 and back to 25 °C) and γ-ε forward transformation through cooling and ε-γ reverse transformation through heating.

The γ/ε interfaces do not have the normal direction to the interface, present in the thermo-elastic martensitic transformations, for example, in the case of SMA based on Ti-Ni. In contrast, ε-martensite increases in the direction parallel to the γ/ε interfaces, and thickening occurs due to the coalescence of nearby thin ε plates. Microstructure analysis showed that ε-martensite has fine lamellar structures involving thin remaining layers and/or high probabilities of stacking defects [44]. Stacking failure inside ε-martensite is a thin plate with a thickness of two atomic layers. The appearance of the microstructure can be associated with the distribution of nucleation sites and the growth of martensite crystals; this increase in martensite occurs due to the displacement of partial Shockley dislocations [45]. The SME consists in the deformed state in which ε-martensite is induced as shown in Figure 3, which subsequently returns to the original γ—austenite shown in Figure 2a by the inverse transformation to heating. Even so, the shape recovery strain in the binary FeMn alloys is tiny [45]. To achieve an optimal SME, a second necessary element should be added, specifically, silicon.

In Figure 3, DSC diagram of C and W samples in the −50 to 200 °C domain (cooling from room temperature to −50 °C, heating to 200 °C and cooling to 25 °C) are presented. In the case of the cast sample, no variation of the thermal flux is observed except for two small variations on cooling around 10 and −10 °C that appear after heating of the cast sample to 200 °C.

The SME of these FeMn-Si-based alloys is closely related to the martensitic transformation induced by deformation of austenite with cubic structure with centered faces in martensite with closed hexagonal structure, the reverse of the phenomenon being possible at subsequent heating. In the wrought sample, Ms temperature is at 35 °C and Mf at 1.5 °C with dissipated heat (ΔH/m) [kJ/kg] of 7.6 associated with the γ → ε transition. The SME will be evaluated in a different article through tensile and bending tests to establish the application potential of this material as an SMA element.

The plastic deformation of the FCC and HCP structures is achieved by different sliding modes with extended dislocation (a), mechanical γ-twinning (b), and martensitic trans-formation γ → ε (c), because of the expansion of stacking defects and their regular or irregular duplication, depending on the relative stability γ/ε. [46]. An important role in determining the mechanisms of plasticity is played by the energy of stacking defects. The DSC result of the wrought state confirms the appearance of the martensitic peak ε (100) at 41.23619° on XRD result.

### 3.2. Electrolyte Solution pH Variation in Contact with Metallic Sample Analysis

Corrosion initially began when the samples were immersed in Ringer’s solution. The oxidation reaction has randomly occurred in several areas of the anodic outer surface, at the grain boundaries, and at the interface between phases; see Equations (1) and (2). The cathodic reaction of the reduction of water that consumed the released electrons fol-lowed (see Equation (3)). Further, the layers of insoluble hydroxides (metal oxides) were formed from free metal ions, which reacted with hydroxide ions (OH−) (see Equations (4) and (5)). In the subsequent visual observations, these hydroxides appeared as a red-brown (Fe_2_O_3_) layer on the top and a black (Fe_3_O_4_ and FeO) layer on the bottom.

**Figure 4:**
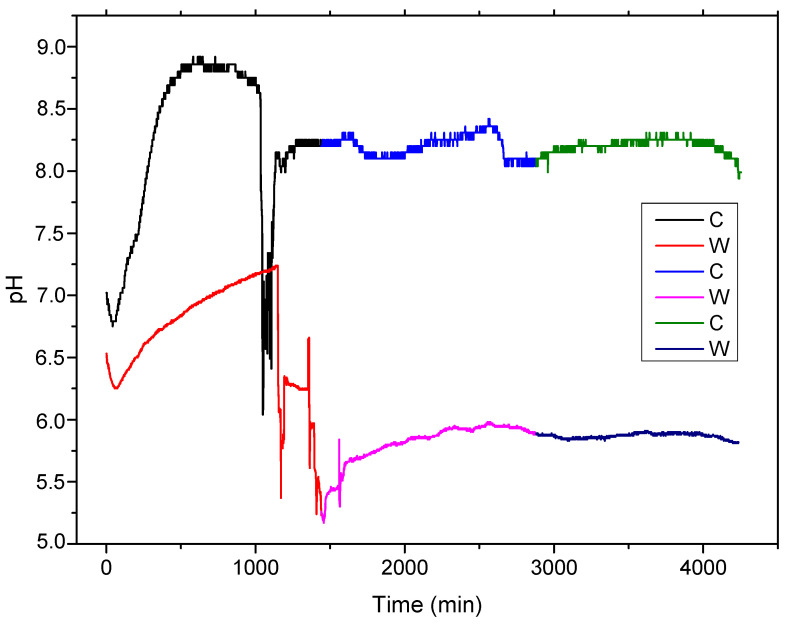
The pH variation during 72 h of immersion at 37 °C of melted and wrought sample.

Based on the fact that the general reaction consumes H^+^ and produces OH^−^, the electrolyte solution pH will increase, enhancing the formation of an Fe(OH)_2_ thin layer on the experimental alloy surface (precipitation reaction). This process characterizes both samples, melted and wrought (see Figure 4), in the first 16–17 h of contact. The porous penetrable layer, in which most of the compounds are oxides, formed on the surface play a protective role for substrate in this time, slowing down new corrosion processes.

Chloride ions compensated for the growth of metal ions under the hydroxide layer by penetrating the metal substrate. The metal chloride formed was then hydrolyzed into hydroxide and free acid; see Equation (6). This decreased the pH value in the pitting pits, and the solution remained neutral. An in vitro static and dynamic degradation was performed [4,5]. The decrease in the solution’s pH was observed after immersion for 16–17 h for both C and W samples, with different rates, approximately 60 to 90 min for melted sample and 500 min, with pH variations based on different areas of breakthrough, for wrought sample. It can be observed that the cast sample of FeMnSi presents a faster corrosion rate than the wrought material, due to a bigger grain structure and main structural defects obtained from melting.

Initial corrosion reaction (a):

Fe→ Fe^2+^ + 2e^−^ (1)

Mn→Mn^2+^ + 2e^−^ (2)

2H_2_O+O_2_+ 4e^−^→4OH^−^ (3)

Formation of hydroxide layers (b):

2Fe^2+^+ 4OH^−^→ 2Fe(OH)_2_ or 2FeO·2H_2_O (4)

4Fe(OH)_2_ +O_2_ + 2H_2_O → 4Fe(OH)_3_ or 2Fe_2_O_3_·6H_2_O (5)

Pitting formation (c):

Fe^2+^+ 2Cl^−^ → FeCl_2_ + H_2_O → Fe(OH)_2_+HCl (6)

Further pH variations can be observed in the subsequent two days (2880 min) for both samples based on passivation and repassivation of the surface in the electrolyte solution [47].

### 3.3. Immersion Experiments Analyses

Immersion experiments present a clearly loose of mass after one or seven days in Ringer’s solution at 37 °C. Mass variation of the samples was recorded for cast and wrought samples, see Table 2, and present an increase in mass after one day based on the compounds formed after the interaction of the material with Ringer’s solution (generally oxides) with a bigger value for cast sample (enhanced corrosion).

The degradation rate presented in Table 2 resulted according to the formula [48]:

(7) $$DR=\frac{8.76\times 10^{4}W}{At\rho }$$

where: *DR* = degradation rate (mm/year); *W* = mass loss (g); *A* = sample area (cm^2^); *t* = time of immersion (h); and *ρ* = metal density (g/cm^3^). This confirms the higher value of corrosion of the cast sample compared with the wrought one.

The sample mass presents a decrease after ultrasound cleaning (in all cases) based on the removal of the compounds formed on the surface through immersion. The stability of the compounds formed on the surface is low even after one day of reactions. After 3 and 7 days before and after ultrasound cleaning, we observed a decrease in the sample mass, more exfoliation of the surface being done during this period, and the corrosion compounds passed in Ringer’s solution. A bigger mass loss is observed in case of the cast sample, meaning a higher corrosion than the wrought sample (around two times higher for the cast sample).

Chemical composition insights of the surface after immersion in Ringer’s solution before and after ultrasound cleaning are given in Table 3 after EDS determination on a 4 mm^2^ surface. In addition to the alloy main elements, respectively, Fe, Mn, and Si, new elements were identified on the surface after the interaction with Ringer’s solution, generally O, C, Cl, and Na. The difference between the chemical composition of the surface before and after ultrasound cleaning are substantial, indicating that most of the compounds are unstable on the surface from the first day of contact with the electrolyte solution. However, after seven days a lower loss of compounds is observed, indicating that the stability of the compounds is better, the interaction with the substrate is higher, and the loss of material occur in larger quantities.

**Figure 5:**
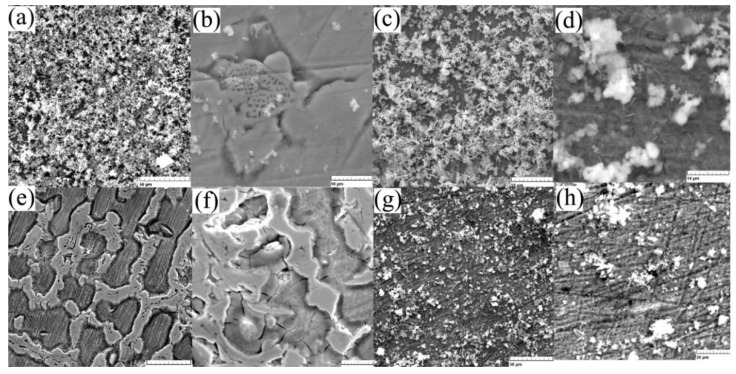
SEM images of the surface: (**a**) C sample after 1 day immersion (1 kx) and (**b**) after ultrasound cleaning (5 kx), (**c**) W sample after 1 day immersion (1 kx) and (**d**) after ultrasound cleaning (5 kx), (**e**) C sample after 7 days immersion (1 kx), and (**f**) after ultrasound cleaning (2 kx), (**g**) W sample after 7 days immersion (1 kx) and (**h**) after ultrasound cleaning (2 kx).

Structural aspects of the surface after immersion tests were taken using scanning electron microscopy, see Figure 5, and present the main aspects of the compounds formed on the surface. A more stable anchoring of the compounds after 7 days is observed from Figure 5e–h, confirming the observations made from chemical composition analyses. After initial immersion (Figure 5a–d), the entire surface is covered by reaction compounds, generally iron and manganese oxides (see Table 3), which are mainly removed from the surface after cleaning. Figure 5b shows the compounds formed at the micro scale as well as the corroded surface of the sample. On the wrought sample a reduced quantity of compounds is observed (see Figure 5g,h) before and after sonication confirming the mass variation quantities given in Table 2.

For the cast state, the corrosion begins at the surface on the first day without the formation of many compounds and more an attack of the structure (Figure 5b,f), and after seven days of immersion a thick layer of compounds is observed on the wrought sample (Figure 5g,h). Considering a low corrosion rate of Fe-based biodegradable materials compared to Mg-based, we can note an advantage of the higher corrosion rate of the cast material.

**Figure 6:**
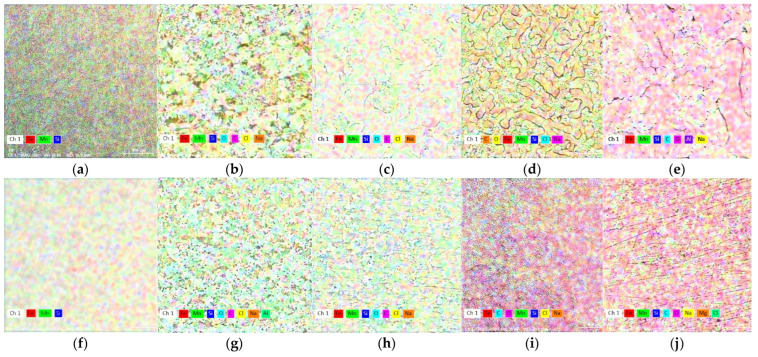
X-ray mapping of chemical elements on the surface. The initial samples, cast (**a**) and wrought (**f**), (**b**); C sample after 1 day immersion and (**c**) after ultrasound cleaning; (**d**) C sample after 7 days immersion and (**e**) after ultrasound cleaning; (**g**) W sample after 1 day immersion and (**h**) after ultrasound cleaning; (**i**) W sample after 7 days immersion and (**j**) after ultrasound cleaning.

In Figure 6, the state of the experimental materials surface is presented through elemental distributions. In the initial state (C and W) no visible differences of chemical elements distribution is observed (Figure 6a,f) so the phase transformations occur with very small modifications of the chemical composition (γ to ε or the reverse).

By addition to FeMn system, silicon replaces iron, contributing to an increase of the Mn:Fe report in the general system composition in the ternary alloy. Silicon is not homogeneously spread in the microstructure and enriched in inter-dendritic regions [49], and will promote a higher degradation rate.

### 3.4. Electro-Chemical Corrosion Resistance

**Figure 7:**
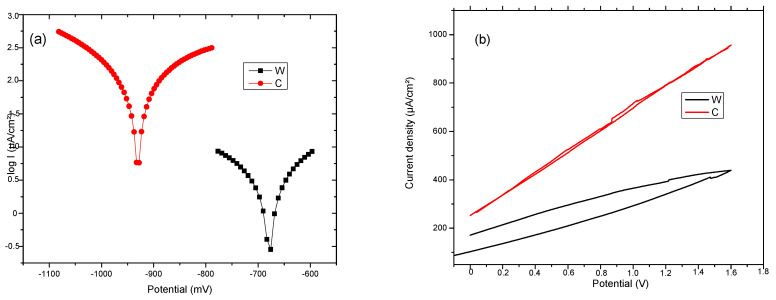
(**a**) Tafel and (**b**) cyclic diagrams for C and W samples.

Complementary data from other sources or sensors must be obtained for a more complete picture of the corrosion process. These complementary data are extracted at the same time as the purchase from the corrosion sensor. Corrosion monitoring is done most accurately by highly sensitive methods that provide an instantaneous signal when the corrosion rate changes. Changes in corrosion potential can give indications of active/passive behavior in alloys. Moreover, the corrosion potential can give fundamental indications on the possibility of corrosion from a thermodynamic perspective (thermodynamic probability of corrosion. The corrosion potential, E_corr_, is a measure of the corrosion tendency of an alloy immersed in an electrolytic environment. Corrosion is evaluated indirectly, from the linear polarization curves, using a Tafel diagram (see Figure 7). The intersection of the linear portions of the anodic and cathodic branches of the polarization curve gives the potential the value of the corrosion potential, E_corr_. Electrochemical properties of the samples C and W were determined by Tafel polarization and EIS measurements. Values of the corrosion parameters, such as corrosion current density (i_corr_), corrosion potential (E_corr_), and protection efficiency (V_corr_), were extracted from the Tafel curves and are presented in Table 4. The (E_corr_) value of Sample W is −678.9 mV and for sample T is −930.7 mV. The shift of the E_corr_ toward negative values implies a higher corrosion resistance for the wrought sample.

The corrosion current density j_corr_ value for W, which is directly proportional to the corrosion rate, is lower than the value for C. This can be attributed to the surface defects on the sample C. Based on the analysis of cyclic polarization diagrams, it can be observed that there are not any significant differences between the two samples. This presents uniform (generalized) corrosion when polarized anodically. Hydrogen is also released at lower potentials, but in tiny quantities for Fe-based alloys.

The FeMnSi system (C and W) exhibits a tendency of active dissolution under anodic polarization conditions, compared to the Fe-Mn alloy [50]. The presence of Si appears to slow down the kinetics of the anodic reaction, a fact suggested by the slope change of the binary alloy from 70 mV/decade to 100 mV/decade in the case of the ternary alloy. A transformation to SiO_2_ may occur due to the release of Mn and Fe ions from the surface, which will cover the surface and thus reduce the dissolution rate, as shown in the diagram of the potential pH of Si-H2O [50]. The process of dissolving Mn and Fe leads to a more alkaline pH and destabilization of surface oxides [50]. Before switching to a controlled mass transfer regime, the slope suddenly increases to over 600 mV from the SCE in the case of the polarization curve of FeMnSi. The increase of degradation rate by Si adding to FeMn system is given by the irregular spread of Si at the grains limits and promotion of areas with different corrosion potential (similar to micro-piles formation).

### 3.5. Electro-Impedance Spectroscopy (EIS) Experiments

The data obtained show that, for these systems, the behavior in solution can be described using a circuit equivalent to a single time constant: a Randles-type circuit, which indicates that the corrosion process occurs on the entire surface of the sample (generalized corrosion) by a single chemical reaction (most likely corrosion of iron with the formation of soluble and insoluble products).

A constant phase element (CPE) was used instead of the Randles circuit capacitance, for a better explanation of the deviation of the Nyquist diagrams from the ideal behavior (a semicircle on the abscissa axis, Zr) due to the change in capacitances with frequency. The impedance of CPE is expressed mathematically with the help of the following relation [51,52,53]:

(8) $$Z_{CPE}=\frac{1}{Q{\left(j\omega \right)}^{n}}$$

where: *Q* is a constant proportional to the active area; <*Q*> = Ω^−1^ s^n^/cm^2^ ≡ S·s^n^/cm^2^, *ω* is theangular frequency (*ω* = 2πf, f is the frequency of the applied alternating current), and j is the imaginary number; *j* = (−1)^½^.

Because of this relationship, the phase angle of the CPE is independent of frequency and has a value of (90°)^n^, which is also the reason why it is called a constant phase element.

The circuit elements have the following meanings: *R_s_*—electrolyte resistance between the working electrode and the reference electrode, *R_ct_*—resistance to charge transfer through the double-electric layer (ct → charge transfer) thus controlling the speed of the corrosion process, and CPE—element of constant phase, which, in theory, would represent the capacity of the double-electric layer (*C_dl_*), but here it has the meaning of an imperfect capacitor (*n* < 1). Imperfections can be mechanical (rough surface) and/or chemical (non-uniform chemical composition).

The effective value of the double-layer capacity can be calculated using the Brug relation [50]:

(9) $$C_{dl}={\left[Q{\left(\frac{1}{R_{s}}+\frac{1}{R_{ct}}\right)}^{n-1}\right]}^{\frac{1}{n}}$$

Using this relationship and the data in Table 5, we obtain: *C_dl_* = 4.9155 × 10^−3^ F/cm^2^.

In Table 5, ε represents the percentage error of evaluation of each circuit element.

From the viewpoint of the values of χ^2^, the circuits R (QR) (QR) and R (CR) (QR) seem much more appropriate than the simple circuit R (QR) (χ^2^~10^−3^), but from the viewpoint of the individual evaluation errors of the circuit elements seems to be more appropriate is the last circuit, in which the percentage errors are insignificant.

The R (CR) (QR) circuit fits the experimental curve very well. The admission of this circuit indicates that the complex layer on the sample surface is forcibly divided into two layers: SDE (electric double layer where the reaction occurs) and another layer. To see if this is so, for the circuit R (QR) (QR) we calculated the global resistance series: R= R_1_ + R_2_ and *Q* global series: *Q* = (Q_1_.Q_2_)/(Q_1_ + Q2) (because Q has character capacitor), and we found R = 165.14 ohm·cm^2^, respectively Q = 7.838 10^−3^ S·s^n^/cm^2^, which are close to the values R = 199.8 ohm·cm^2^, respectively Q = 9.424 10^−3^ S·s^n^/cm^2^ found for circuit R (Q). The differences could be attributed to large errors in the evaluation of the parameters with the R (QR) (QR) circuit.

**Figure 8:**
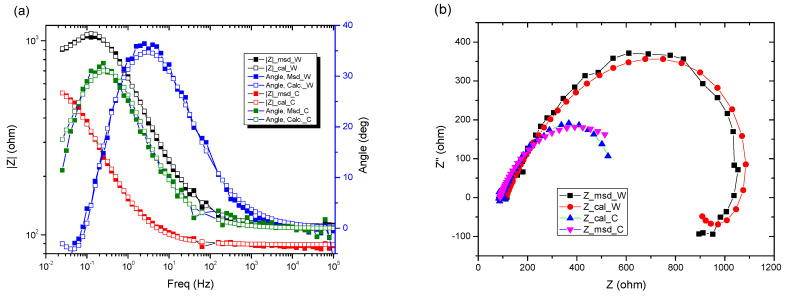
Electrochemical measurements of tested samples for (**a**) Bode plots, (**b**) Nyquist plots.

In the Nyquist diagram, the experimental curve shows a negative loop in the low frequency range. This distortion is most often attributed to an inductive behavior of the electrochemical system due to the process of adsorption of intermediates on the surface of the electrode (sample). Here, it would most likely be adsorption of Fe or Fe_2_O_3_. For data simulation, it is necessary to use an equivalent circuit with inductance (Figure 8).

In the circuit used in Table 6, R_s_, Q, and R_ct_ have the same meaning as in the previous circuits; Q and R_ct_ characterize the double-electric layer that controls the corrosion rate, in the Nyquist diagram being represented by the capacitive loop in the dolmen of high frequencies. In the same circuit W represents an inductance and R_L_ is the total resistance of the adsorbed particles on the surface unit of the sample.

Note that the adsorbed intermediate does not form a compact or porous layer but has the appearance of islands on the surface of the sample, a structure that favors a relaxation or reduction of sample capacity in the corrosion process (the effective active surface of the sample is reduced).

In this case, the effective capacity of the electric double layer is calculated using the modified Brug relation:

(10) $$C_{dl}={\left[Q{\left(\frac{1}{R_{s}}+\frac{1}{R_{p}}\right)}^{n-1}\right]}^{\frac{1}{n}}$$

where *R_p_* is the polarization resistance: *R_p_* = ((R_ct_·R_L_)/R_ct_ + R_L_)

This circuit describes a system in which a single uniform corrosion reaction takes place, without the involvement of other types of processes such as diffusion or adsorption.

Moreover, both the Nyquist and Bode curves indicate that the experimental data are suitable for a circuit with a single time constant: the R (CR) circuit.

## 4. Conclusions

An iron-based SMA was realized thorough a classical melting method with a new chemical composition for possible applications in the medical field as biodegradable material. The analysis of the experimental results led to the following conclusions:

- A smart Fe-based biodegradable alloy can be applied to the medical field with a proper thermo-mechanical treatment to modify the transformation temperatures;
- Reactions between the alloy and electrolyte modify the pH of the environment;
- The cast sample presents a higher corrosion rate than the wrought one based on the microstructure arrangement, microstructural defects, or grain dimension being suitable for certain medical applications;
- The corrosion current density j_corr_ value for W, which is directly proportional to the corrosion rate, is lower than the value compared for C. This can be attributed to the surface defects on the sample C;
- Based on the analysis of cyclic polarization diagrams, it can be observed that there is not any significant difference between the two samples. They present uniform (generalized) corrosion when polarized anodically;
- The data obtained from EIS experiments show that, for these systems, the behavior in solution can be described by using a circuit equivalent to a single time constant; a Randles-type circuit, which indicates that the corrosion occurs on the entire surface of the sample (generalized corrosion) by a single chemical reaction (most likely corrosion of iron with the formation of soluble and insoluble products).

## Figures and Tables

**Table 1:** Experimental results after chemical composition analysis for the initial cast sample and wrought state (average values after five determinations on different 1 mm^2^ areas).

Elements/State	Fe		Mn		Si	
	wt%	at%	wt%	at%	wt%	at%
Cast	82.29	79.11	13.88	13.56	3.83	7.32
Wrought	82.16	79.33	14.47	14.2	3.37	6.48
EDS error %	0.06		0.02		0.03	

Standard deviation (StDev): Fe ±0.15, Mn: ±0.1 and Si: ±0.05.

**Table 2:** The results of the masses of the experimental samples after 1, 3, and 7 days of immersion in Ringer’s solution (five repetitive mass determinations were done on the same sample) and the corresponding degradation rate.

Sample	1 Day		3 Days		7 Days	
	Cast Sample (C)	Wrought Sample (W)	Cast Sample (C)	Wrought Sample (W)	Cast Sample (C)	Wrought Sample (W)
Initial mass (mg)	3182.7	563.1	2756.6	471.9	2989.7	513.8
Mass after immersion (mg)	3184.3 (+1.6)	563.3 (+0.2)	2756.3 (−0.3)	465.7 (−6.2)	2984.3 (−5.4)	511.5 (−2.3)
Mass after ultrasonic cleaning (mg)	3182.0 (−0.7)	562.6(−0.5)	2752.8 (−3.8)	463.9 (−8.0)	2983.9 (−5.8)	510.5 (−3.3)
DR (mm/year)	0.088	0.084	0.159	0.451	0.104	0.080

Standard deviation: ±0.1 mg.

**Table 3:** The chemical composition of the FeMnSi alloy after 1 day and 7 days of immersion in Ringer’s solution and after ultrasonic cleaning after each period for both cast and wrought samples.

El./Samples			Fe		Mn		Si		O		C		Cl		Na	
			wt%	at%	wt%	at%	wt%	at%	wt%	at%	wt%	at%	wt%	at%	wt%	at%
1 day (I)	C	I	56.16	29.39	8.75	4.66	1.86	1.93	22.02	40.23	8.39	20.42	0.49	0.41	2.33	2.96
		I + UC	67.55	42.43	12.3	7.85	2.78	3.47	5.89	12.9	11.37	33.2	0.05	0.04	0.07	0.1
	W	I	48.77	22.01	5.74	2.63	1.23	1.1	33.53	52.83	9.82	20.62	0.51	0.37	0.4	0.44
		I + UC	53.11	25.8	8.38	4.14	1.77	1.71	25.06	42.49	11.32	25.57	0.32	0.25	0.05	0.06
7 days (I)	C	I	57.92	29.92	10.29	5.4	2.32	2.39	13.54	24.41	15.65	37.6	0.15	0.12	0.13	0.17
		I + UC	56.85	28.53	9.78	4.99	1.94	1.94	14.56	25.5	16.59	38.7	-	-	0.28	0.34
	W	I	45.57	19.99	7.1	3.17	1.57	1.37	32.53	49.83	11.87	24.23	0.12	0.08	1.24	1.32
		I + UC	52.81	25.16	7.7	3.73	2.05	1.94	21.73	36.13	14.13	31.3	0.21	0.16	1.23	1.42
EDS detector error %			1.54		0.31		0.17		4.04		4.23		0.07		0.18	

C: cast, W: wrought, I: after immersion, I + UC: after immersion and ultrasound cleaning. StDev: Fe: ±0.9, Mn: ±0.5, Si: ±0.22, O: ±0.2, C: ±0.1, Cl: ±0.1, Na: ±0.1.

**Table 4:** Linear Tafel parameters for C and W samples.

Sample	E_corr_ mV	b_a_ mV	b_c_ mV	R_p_ ohm·cm²	J_corr_ mA/cm²	V_corr_ mm/Year
C	−930.7	324.8	−224.3	388.57	2.17	132.9
W	−678.9	95.2	−156.7	1000.42	0.11	24.47

**Table 5:** The values of the equivalent circuit for sample C.

R(QR)		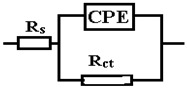					
**R_s_ (ohm·cm^2^)**		**10^3^. Q (S·s^n^/cm^2^)**		**n**		**R_ct_ (ohm·cm^2^)**	**10^3^.χ^2^**
29.8		9.424		0.684		199.8	1.33
ε (%): 0.6998		2.135		1.705		3.828	
R(QR)(QR)		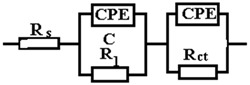					
**R_s_ (ohm·cm^2^)**	**Q_1_** **(S·s^n^/cm^2^)**	**n_1_**	**R_1_ (Ω·cm^2^)**	**Q_2_ (S·s^n^/cm^2^)**	**n_2_**	**R_2_ (Ω·cm^2^)**	**10^3^.χ^2^**
29.26	0.01193	0.610	73.63	0.02281	0.9851	91.51	0.440
ε: 0.6998	13.06	3.691	129.8	100.2	17.1	90.7	
R(CR)(QR)		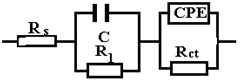					
**R_s_ (ohm·cm^2^)**	**C (F/cm^2^)**	**R_1_ (Ω·cm^2^)**		**Q (S·s^n^/cm^2^)**	**n**	**R_ct_ (Ω·cm^2^)**	**10^3^.χ^2^**
29.27	0.02468	85.33		0.01175	0.612	80.47	0.438
ε: 0.6437	13.51	13.55		6.191	3.33	21.8	

The authors tried three circuits that seem to be suitable for interpretation, with the values presented in Table 5.

**Table 6:** The values of the equivalent circuit for sample C.

(R(QR(LR))		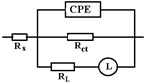				
R_s_ (ohm·cm^2^)	Q (S·s^n^/cm^2^)	n	R_ct_ (ohm·cm^2^)	L (Henry·cm^2^)	R_L_ (ohm·cm^2^)	10^3^.χ^2^
36.29	0.001161	0.665	424.1	1881	655.8	1.30
ε%: 1.737	4.379	1.829	4.237	12.94	7.04	

## Data Availability

All data presented in this study are contained within the article.
